# Obstetric and neonatal outcomes, antiseizure medication profile, and seizure types in pregnant women in a vulnerability state from Brazil

**DOI:** 10.1371/journal.pone.0291190

**Published:** 2024-04-01

**Authors:** Magnúcia de Lima Leite, Tatiana Natasha Topocov, Tales Lyra de Oliveira, Daniel dos Santos Almeida, Sandra Regina Mota Ortiz, José Claudio da Silva

**Affiliations:** 1 Universidade Estadual de Ciências da Saúde Alagoas (UNCISAL), Maceió, AL, Brazil; 2 Universidade de São Paulo (USP), São Paulo, SP, Brazil; 3 Universidade Municipal de São Caetano do Sul (USCS), São Paulo, SP, Brazil; 4 Universidade São Judas Tadeu (USJT), São Paulo, SP, Brazil; 5 Faculdade de Medicina do Centro Universitário(CESMAC), Maceió, AL, Brazil; 6 PPGSF/RENASF/FIOCRUZ/UNCISAL, Maceió, AL, Brazil; Belgrade University Faculty of Medicine, SERBIA

## Abstract

This retrospective cohort study described the obstetric and neonatal outcomes, antiseizure medication (ASM) use, and types of seizures in pregnant women with epilepsy (PWWE). Data collected from the medical records of 224 PWWE aged < 40 years with controlled or refractory seizures and 492 pregnant women without epilepsy (PWNE) control group from high-risk maternity hospitals in Alagoas between 2008 and 2021 were included in this study. The obstetric and neonatal outcomes observed in PWWE were pregnancy-related hypertension (PrH) (18.4%), oligohydramnios (10.3%), stillbirth (6.4%), vaginal bleeding (6%), preeclampsia (4.7%), and polyhydramnios (3%). There was a greater likelihood of PrH in PWWE with generalized tonic-clonic seizures (GTCS) and that of maternal intensive care unit (ICU) admissions in those with GTCS and status epilepticus, and phenytoin and lamotrigine use. PWWE with GTCS had a higher risk of stillbirth and premature delivery. PWWE with status epilepticus were treated with lamotrigine. Phenobarbital (PB) with diazepam were commonly used in GTCS and status epilepticus. Total 14% patients did not use ASM, while 50.2% used monotherapy and 35.8% used polytherapy. Total 60.9% of patients used PB and 25.2% used carbamazepine. This study described the association between the adverse obstetric and neonatal outcomes and severe seizure types in PWWE.

## Introduction

According to the International League Against Epilepsy (2014), epilepsy is a brain dysfunction characterized by a maintained predisposition of generating and propagating epileptic seizures with an occurrence of two or more unprovoked seizures within 24 hours. Basically, seizure types are classified as focal onset (motor, nonmotor onset) and focal to bilateral tonic-clonic; generalized onset motor (tonic-clonic, other motor), nonmotor (absence), and seizures of “unknown onset,” which may be referred to as “unclassified” or may have additional features, including motor (tonic-clonic, other motor) and nonmotor [1–3].

Approximately 15 million women with epilepsy, globally, are of childbearing age. Most of these women require effective and safe pharmacological treatment for seizure control during pregnancy because uncontrolled seizures can be harmful to both the fetus and pregnant woman. Most women with epilepsy have normal pregnancies; however, there are fetal and obstetric risks associated with epilepsy treatment during pregnancy, such as pregnancy-related hypertension (PrH), preeclampsia, vaginal bleeding, oligohydramnios, polyhydramnios, miscarriage, negative effects on fetal growth, increased risks of major congenital malformations (MCM), stillbirth, and adverse effects on neurocognitive and behavioral development [4–6].

Regarding obstetric complications in PWWE, a Finnish population-based cohort study from 1989–2000 did not show these results [7], while Norwegian population-based cohorts from 1999–2005 and 2004–2012 and another United States population-based cohort from 2007–2011 in 20% of community hospitals showed a significantly increased risk of mild preeclampsia due to PrH and delivery of < 34 weeks of gestation and severe postpartum hemorrhage along with preterm delivery of < 37 weeks, respectively [5, 8, 9]. Overall, complications include hypertensive disorders, delivery conditions, bleeding during gestational period, and infectious diseases [10].

In PWWE, the main causes of increased seizures are still unclear, although there is a multifactorial causality [11]. Physiological changes during pregnancy alter the pharmacokinetics of ASM, resulting in low bioavailability of the drug and requiring dose adjustments; this is possibly due to hormonal changes, sleep deprivation, and psychosocial stress, although the latter factors have not been studied systematically [12]. Recent studies have revealed that women without records of epileptic seizures before 9 months to 1 year of pregnancy had an 84–92% chance of being seizure-free, and the presence of epileptic manifestations up to the month before pregnancy was the main predictor of seizure recurrence during pregnancy [13–15].

The risk of MCM in children of women with epilepsy depends on the type, number, and dosage of drugs administered. Some recent ASMs, such as levetiracetam (LEV) and lamotrigine (LTG), are increasingly being used for seizure treatment, except for topiramate (TPM) [16]. Another cohort study in Norway was conducted from 1999 to 2011, and it compared 2,600 children of WWE exposed to MACs and 771,412 children of WWE without MAC use. It was revealed that TPM is associated with a considerable risk of microcephaly (11.4% vs. 2.4%, odds ratio (OR): 4.8, 95% confidence interval (CI): 2.5–9.3) and low birth weight (24.4% vs. 8.9%, OR: 3.1, 95% CI: 1.9–5.3) [17]. With regard to neurodevelopment, one of the first studies showed that a group of nine preschool children (3–6 years, 11 months) was exposed in utero to TPM monotherapy, and they were compared to a control group of 18 children. The skills of the children were investigated, and results showed that there was a significant difference in motor function, cognition, and behavior between the exposed children and controls [18].

In the first 6 weeks of pregnancy, which is the period wherein seizures are most likely to occur, LEV and LTG are recommended, since they have a low risk of malformations and, in some cases, are similar to those in the general population [4]. As recommended by the National Institute for Health and Care Excellence (2021), treatment choices should consider seizure type and other characteristics, such as age, sex, and comorbidities. The clinical management of epilepsy should include the following measures during pregnancy to minimize the occurrence of MCM and protect maternal health: 1) Frequent monitoring of serum concentrations of ASM and 2) folic acid supplementation [19]. Morrow et al. (2009) and EURAP group could not reach a conclusion regarding the efficacy of folic acid in preventing neural tube diseases or other MCMs associated with ASM [6–20].

This pioneering study in the northeastern region of Brazil had limited health care resources. Approximately Approximately 35,2% of the general population in Alagoas has not education, and higher levels of unemployment 10,65% [21]. Additionally, the Brazilian National Household Sample Survey in 2022 reported that approximately 54% of the general population in Alagoas received less than one minimum wage, which explained our results; this indicated severe per capita inequality in the country with the lowest average income in the northeastern population than that of others [22]. However, this study aimed to describe and analyze the obstetric and neonatal outcomes and determine the most prescribed ASM and the types of seizures in PWWE from high-risk pregnancy reference centers in Alagoas. This is to help the Unified Health System (SUS) in the distribution of more effective ASM to be used in the control of epileptic seizures during pregnancy, ensuring the reduction of adverse obstetric and fetal outcomes.

## Materials and methods

This study was approved by the Research Ethics Committee of the Universidade de São Paulo (USP), São Paulo, Brazil (protocol number: 4.604.088) and the co-participating institutions, the Universidade Estadual de Ciências da Saúde de Alagoas (UNCISAL) and the Universidade Federal de Alagoas (UFAL) (protocol numbers: 4.491.415 and 4.422.629, respectively). Due to the retrospective nature of the study, these three institutions waived the need to obtain informed consent.

This was a retrospective cohort study with data collected from physical and electronic medical records of PWWE and pregnant women with no epilepsy (PWNE), the control group, from 2008 to July 2021. The participants were exempted from signing an informed consent form, and their anonymity was guaranteed. The study sample comprised PWWE/PWNE aged ≤ 40 years. Data collection was performed in the prenatal and prepartum clinics of the Hospital Universitário Professor Alberto Antunes in Maceió, Maternidade Escola Santa Mônica in Maceió, Espaço Nascer in Arapiraca, and Hospital Regional Dr. Clodolfo Rodrigues de Melo in Santana do Ipanema, which are the reference institutions for the care of PWWE in the state of Alagoas. Both groups of pregnant women were recruited from the same high-risk referral center that provides assistance to pregnant women. The control group had a poor obstetric history, such as abrupt rupture of amniotic fluid bag (ICD-10 O42) or the presence of a comorbidity not previously related to the research outcomes, such as urinary tract infection or vaginal infection. To confirm the diagnosis of epilepsy, the International Classification of Diseases (ICD-10) codes, G40.0–G40.8, were used, and healthy pregnant women (control group) were selected based on ICD-10 numbers: O23.4 and O23.5 comorbidity related above and absence of epilepsy ICD-10.

For descriptive analyses, categorical variables were presented as frequencies, and continuous variables were presented as means and standard deviations. Chi-square tests and multivariate logistic regression were performed to verify the association between epilepsy diagnosis and ASM with variables related to obstetric and neonatal outcomes. The results are presented using crude and age-adjusted ORs and their respective 95% CIs. To verify the differences between the groups for continuous variables, t-tests were performed for independent samples. An α value of 5% was used for all analyses performed using the statistical software R (v.3.6.1; R Foundation for Statistical Computing, Vienna, Austria).

## Results

The pregnant women with epilepsy (PWWE) were 0.52% (n = 234 /44,927) of the total number of pregnant women at high-risk pregnancy referral centers in the state of Alagoas. Our study included total 726 pregnant women, with 32.2% of PWWE (n = 234) and 67.8% of PWNE) (n = 492), aged 24.94 ± 6.25 years and 23.98 ± 6.89 years, respectively (p = 0.07). Most of the pregnant women (58.2%, p < 0.01) were born in the countryside. Most PWWE were single (47.3%), 7% (p < 0.01) illiterate, and 76.9% were housewives. Among the PWWE, 74.6% (p < 0.01) were more likely to have a cesarean delivery and 21.9% were more likely to have a miscarriage (p < 0.01) (Table 1).

**Table 1:** Comparison of average socio-demographic and obstetric outcome variables between pregnant women with epilepsy(PWWE) (n = 224) and pregnant women without epilepsy (PWNE) (n = 492).

Variables	PWWE	PWNE	P value
	(n = 224)	(n = 492)	
**Age**	**N/%**	**N/%**	0,07
Mean and standard Deviation	24,94 (± 6.25)	23.98 (± 6.89)	
**Race**			0,17
Non Tanned (n = 84)	25 (11,4%)	59 (12,2%)	
Tanned (n = 620)	195 (88.6%)	425 (87.8%)	
**Previous births**			0,58
Primipara (n = 292)	87 (39.7%)	205 (41.9%)	
Multipara (n = 416)	132 (60.3%)	284 (58.1%)	
**Origin**			<0.01
Maceió	92 (41,8%)	-259 (52,7%)	
Countryside	128 (58,2%)	232 (47,3%)	
**Education**			<0.01
Illiteracy	15 (7,0%)	14 (3,0%)	
1–8 years of study	112 (52,1%)	284 (60,7%)	
**Variables**	**PWWE (n = 224)**	**PWNE (n = 492)**	**P value**
	
9 to 12 years of study	80 (37,2%)	165 (35,3%)	
Above 12 years	8 (3,7%)	5 (1,0%)	
**Occupation**			0,28
Housewives	170(76,9%)	353 (72,2%)	
Students	18 (8,2%)	58 (11,8%)	
Others professions	33 (14,9%)	78 (16,0%)	
**Marital status**			0,58
Single	104 (47,3%)	193 (39,5%)	
Married	46 (20,9%)	76 (15,5%)	
Stable union	66 (30%)	214 (43,8	
Divorced/widow	4 (1,8%)	6 (1,2%)	
**Delivery**			<0,01
Vaginal	52 (25,4%)	430 (87,4%)	
Cesarean	153 (74,6%)	62 (12,6%)	
**Miscarriage**			<0,01
No	171 (78,1%)	423 (86,5%)	
Yes	50 (21,4%)	67 (13,6%)	

Source: author’s own production.

A significantly higher difference was observed in PWWE in terms of adverse obstetric and neonatal outcomes. Univariate logistic regression analysis of the obstetric and neonatal outcomes between PWWE/PWNE was performed, and a positive association was observed in PrH (OR = 6.29, 95% CI = 3.50–11.30), vaginal bleeding (OR = 2.54, 95% CI = 1.15–5.59), preeclampsia (OR = 8.04, 95% CI = 2.22–29.10), oligohydramnios (OR = 4,57, 95% CI = 2.24–9.31), polyhydramnios (OR = 7.55, 95% CI = 1.55–36.65), miscarriage (OR = 1.75, 95% CI = 1.16–2.63), and stillbirth (OR = 11.16, 95% CI = 2.22–29.10) (Table 2).

**Table 2:** Descriptive and logistic regression analysis of obstetric and neonatal outcomes between PWWE (n = 234) and PWNE (n = 492).

**Outcomes**	**Epilepsy**				**p-value**	**Univariate Analysis**		**Multivariate Analysis adjusted for age, type of delivery, marital status, place of birth**	
	**Yes (n = 234)**	**No (n = 492)**					
	**N**	**%**	**N**	**%**		**OR**	**95% CI**	**adjusted OR**	**95% CI**
**PrH**	43	18.4	17	3.5	<0.01	6.29	3.50; 11.30	5.67	2.64; 12.17
**Vaginal bleeding**	14	6.0	12	2.4	0.03	2.54	1.15; 5.59	2.42	0.83; 7.05
**Preeclampsia**	11	4.7	3	0.6	<0.01	8.04	2.22; 29.10	6.48	1.33; 31.60
**Eclampsia**	8	3.4	0	0.0	<0.01	NA	NA	NA	NA
**Oligohydramnios**	24	10.3	12	2.4	<0.01	4.57	2.24; 9.31	1.65	0.64; 4.22
**Outcomes**	**Epilepsy**		**No Epilepsy**		**p-value**	**Univariate Analysis**		**Multivariate Analysis adjusted for age, type of delivery, marital status, place of birth.**	
	**N**	**%**	**N**	**%**		**OR**	**95%CI**	**adjusted OR**	**95% CI**
**Polyhydramnios**	7	3.0	2	0.4	<0.01	7.55	1.55; 36.65	7.06	1.10; 45.07
**Miscarriage**	51	21.8	67	13.6	<0.01	1.78	1.19–2.67	1.47	0.85–2.54
**Depression**	12	5.1	0	0.0	<0.01	NA	NA	NA	NA
**Maternal ICU**	32	13.7	0	0.0	<0.01	NA	NA	NA	NA
**Malformation**	5	2.1	6	1.2	0.34	1.76	0.53; 5.85	0.78	0.18; 3.38
**Stillbirth**	15	6.4	3	0.6	<0.01	11.16	3.20; 38.94	1.53	0.10; 22.35
**Respiratory distress**	30	12.8	57	11.6	0.62	1.12	0.69; 1.80	1.34	0.70; 2.55
**Neonatal heart disease**	7	3.0	0	0.0	<0.01	NA	NA	NA	NA
**Neonatal ICU**	7	3.0	13	2.6	0.81	1.13	0.44; 2.88	1.27	0.34; 4.73
**PrematureDelivery <37 weeks**	53	24.1	98	20.0	0.23	1.27	0.87; 1.86	1.45	0.86; 2.45
**Low birth weight**	43	19.7	111	22.7	0.43	0.38	0.56; 1.24	1.00	0.59; 1.70
**PrH. Vaginal bleeding Preeclampsia/ eclampsia**	58	24.8	30	6.1	<0.01	5.07	3.16; 8.15	4.65	2.48; 8.72
**MCM and Stillbirth**	19	8.1	9	1.8	<0.01	4.74	2.11; 10.65	0.60	0.16; 2.17

Source: author’s own production

NA: It was not possible to perform this analysis.

**Fig 1:**
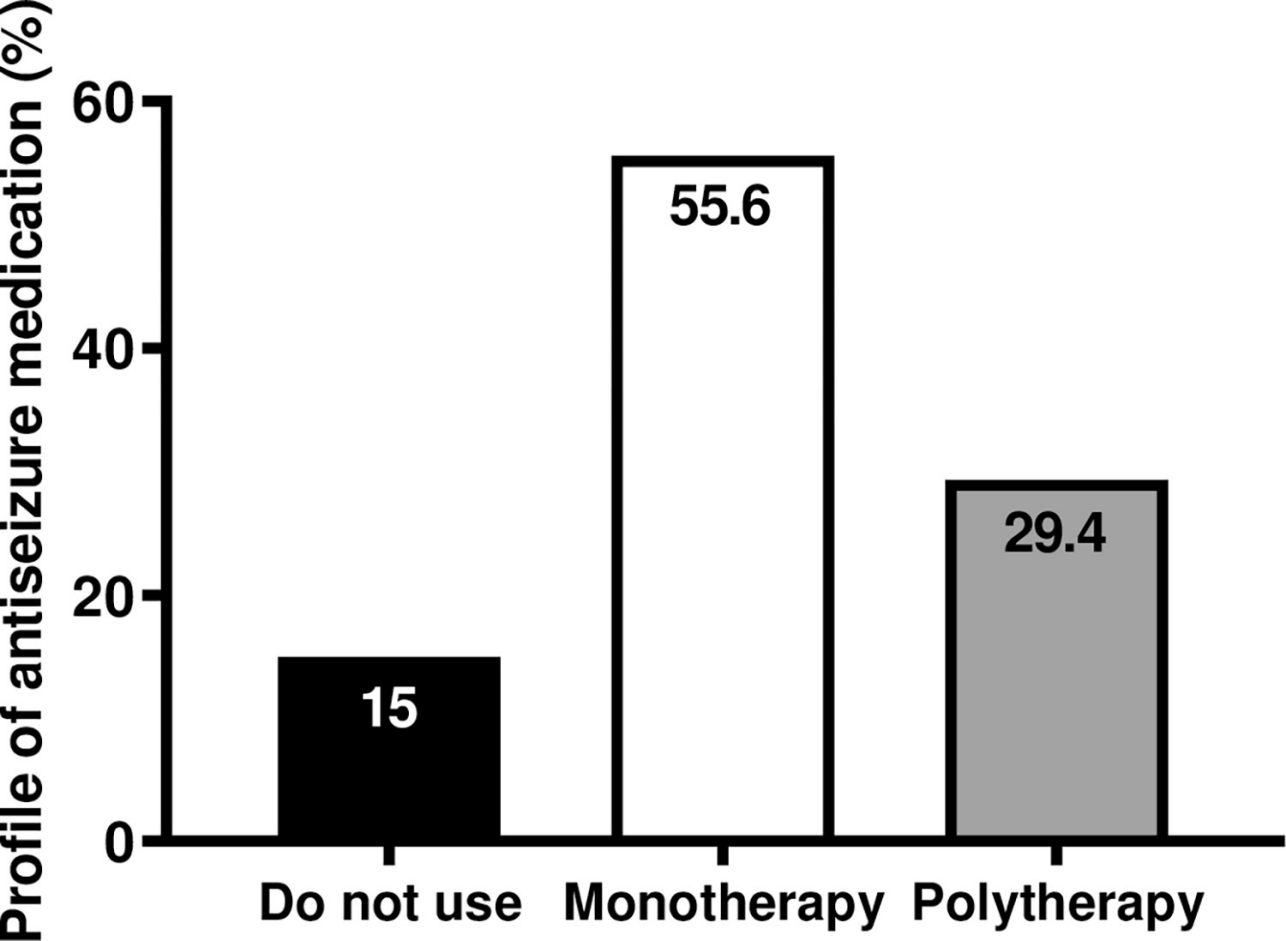
Profile of antiseizure medication. Presenting descriptive analyses with categorical variables as frequencies. Pharmacological treatment most commonly used by PWWE observed that only 15% did not use ASM, 55.6% used monotherapy, and 29.4% used polytherapy.

The commonly used pharmacological treatment in PWWE was analyzed, and we observed that only 14% (n = 32/229) of pregnant women were not prescribed ASM, 50.2% (n = 115/229) were prescribed monotherapy, and 35.8% (n = 82/229) were prescribed polytherapy (Fig 1). Phenobarbital (PB) was the most prescribed drug (60.9%, n = 70/115), followed by carbamazepine (CBZ) (25.2%, n = 29/115) (Table 2), and other drugs in polytherapy (S1 Table).

With regards to pharmacological treatment most commonly used in PWWE, it was observed that only 15% did not use ASM, while 55.6% used monotherapy and 29.4% used polytherapy (Fig 1).

**Fig 2:**
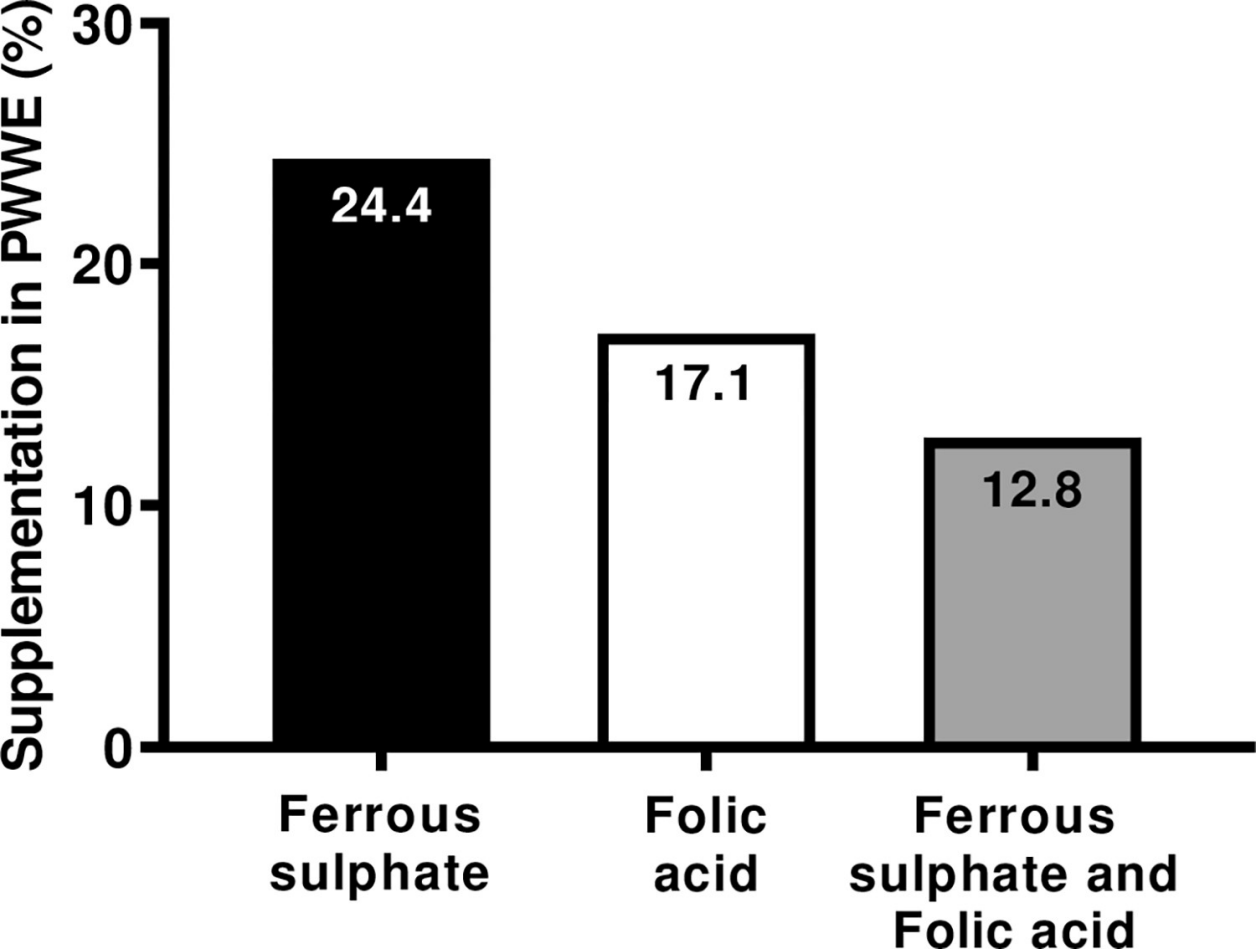
Supplementation used by pregnant women with epilepsy. Observed that only 17.1% of PWWE were prescribed folic acid and 24.4% were prescribed ferrous sulphate supplementation; 12,8% used both.

We found that only 17.1% (n = 40/234) of PWWE were prescribed folic acid and 24.4% (n = 57/234) were prescribed ferrous sulfate supplementation; 12.8% (n = 30/234) used both (Fig 2).

Association tests between ASM in monotherapy and obstetric outcomes revealed that PWWE who were prescribed Phenytoin (PHY) or LTG had a higher chance of clinical worsening with the need for ICU admission (OR = 17.75, 95% CI = 1.95–160.77 and OR = 7.10, 95% CI = 1.03–48.64, respectively). PWWE who were prescribed only PHY had a higher chance of developing preeclampsia or eclampsia (OR = 14.0, 95% CI = 1.61–121.36) (Table 3).

**Table 3:** Profile of ASM used in monotherapy and associated obstetric outcomes (n = 115).

Monotherapy ASM	N	%	PrH	Oligodra mnios	Abortion	Obstetric ICU	Preeclampsia or eclampsia
			OR [95% CI]	OR [95% CI]	OR [95% CI]	OR [95% CI]	OR [95% CI]
**Phenobarbital**	70	60,9	1.00 [0.39; 2.55]	0.77 [0.27; 2.12]	1.03 [0.40; 2.61]	0.11 [0.02; 0.51]	0.35 [0.10; 1.16]
**Valproic acid**	7	6,1	NA	4.65 [0.94; 22.87]	1.93 [0.33; 11.23]	NA	NA
**Carbamazepine**	29	25,2	1.05 [0.37; 3.00]	1.17 [0.37; 3.62]	0.54 [0.16; 1.74]	1.80 [0.48; 6.68]	1.36 [0.38; 4.83]
**Phenytoin**	3	2,6	2.04 [0.17; ‘23.59]	NA	1.89 [0.16; 21.78]	22.88 [1.88; 277.52]	18.36 [1.53; 219.23]
**Diazepam**	1	0,9	NA	NA	NA	NA	NA
**Lamotrigine**	5	4,3	2.82 [0.44; 17.99]	NA	2.60 [0.41; 16.56]	19.12 [2.78; 131.56]	6.00 [0.90; 39.89]

source: author’s own production

Reference: no outcomes presents.

NA: It was not possible to perform this analysis.

**Fig 3:**
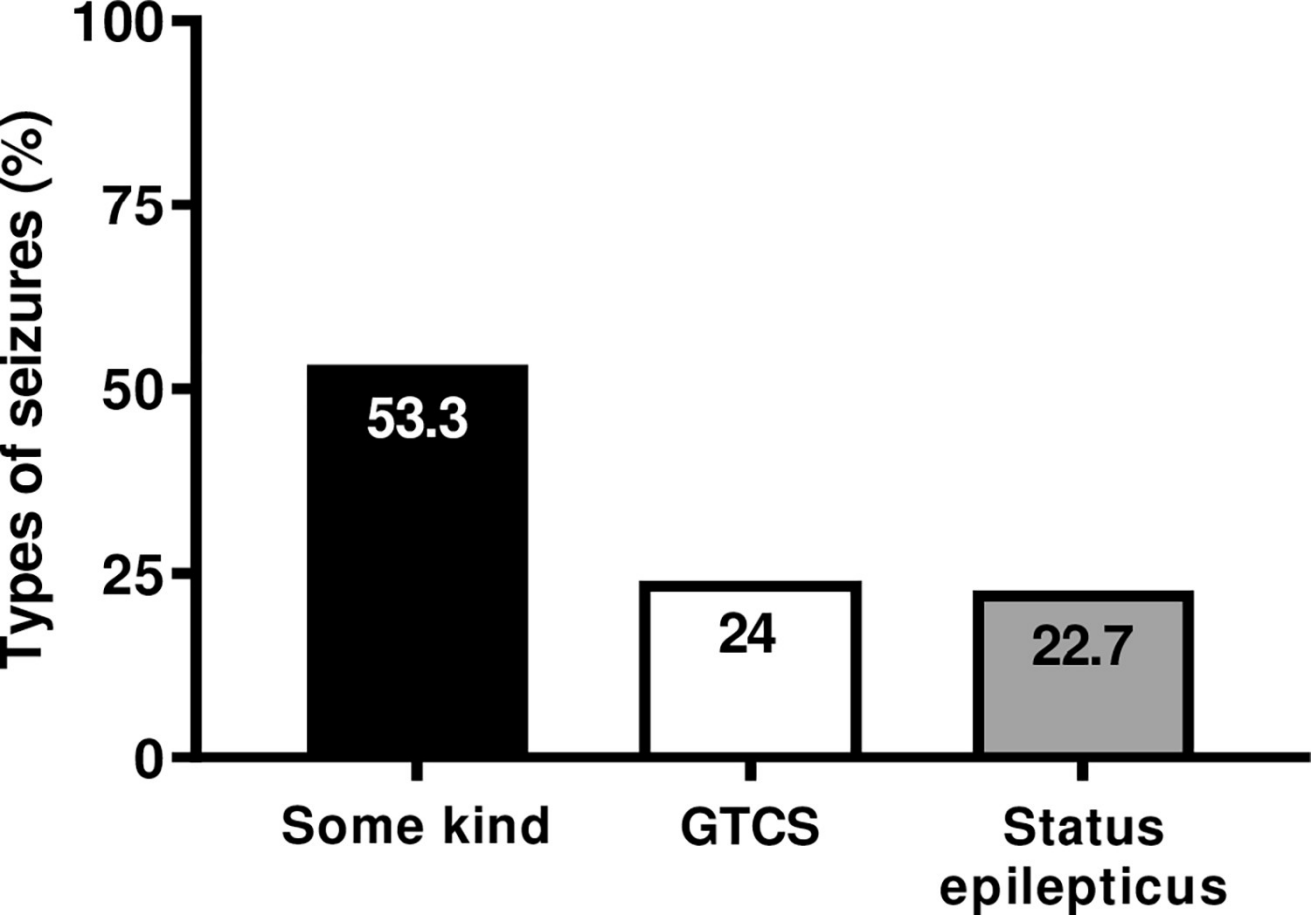
Profile of seizures types. The profile of seizure types revealed that 53.3% had focal seizure, 24% of which were GTCS, and approximately 22.7% evolved to status epilepticus.

Analysis of the profile of seizure types revealed that 53.3% (n = 122/229) of the participants had focal seizure, 24% (n = 55/229) had tonic-clonic generalized (GTCS), and approximately 22.7% (n = 52/229) evolved to status epilepticus (Fig 3).

Correlation of obstetric and neonatal outcomes with types of seizures revealed that PWWE with GTCS had a higher chance of developing PrH and those with GTCS and status epilepticus had a higher chance of ICU admission. PWWE with GTCS had a higher chance of stillbirth and those with status epilepticus had higher chance of premature deliveries (Table 4).

**Table 4:** Regression analysis between obstetric and neonatal outcomes and seizures types (n = 229).

Outcomes Obstetric and neonatal	Seizure type		
	Focal	GTCS	Status epilepticus
	OR [95%CI]	OR [95% CI]	OR [95% CI]
**PrH**	1.00	8.05 [3.47; 18.64]	2.34 [0.89;6.16]
**Vaginal bleeding**	1.00	0.38 [0.08; 1.77]	0.19[0.02; 1.57]
**Preeclampsia**	1.00	NA	NA
**Eclampsia**	1.00	NA	NA
**Oligohydramnios**	1.00	0.31 [0.88; 1.10]	0.10 [0.01; 0.81]
**Polyhydramnios**	1.00	NA	0.93 [0.17; 4.98]
**Miscarriage**	1.00	0.79 [0.35; 1.77]	0.95 [0.43; 2.11]
**Maternal ICU**	1.00	11.73 [2.44; 56.37]	37.50 [8.32; 168.84]
**MCM**	1.00	NA	NA
**Stillbirth**	1.00	3.41 [1.03; 11.28]	0.93 [0.17.4.98]
**Respiratory distress**	1.00	1.12 [0.42; 2.96]	1.40 [0.54; 3.57]
**Neonatal ICU**	1.00	NA	1.18 [0.20; 6.65]
**Neonatal heart disease**	1.00	1.11 [0.19; 6.26]	0.57 [0.06; 5.30]
**Premature**	1.00	1.55 [0.71; 3.42]	2.22 [1.05; 4.69]
**Delivery<37 weeks**			
**Low birth weight**	1.00	0.93 [0.38; 2.29]	1.60 [0.73;3.53]

Reference: Some type of crisis.

NA: It was not possible to perform this analysis.

The univariate logistic regression test of seizure type and ASM showed that PWWE with status epilepticus had higher chance of using LTG (OR = 21.91, 95% CI = 2.07–231.60). PB with diazepam was the most commonly used drug by PWWE for GTCS and status epilepticus (OR = 12.04, 95% CI = 1.43–101.47) (Table 5).

**Table 5:** Logistic regression analysis of types of ASM with seizure type (n = 229).

Drug Treatment	Seizure type		
	Focal	GTCS	Status epilepticus
	OR [95% CI]	OR [95% CI]	OR [95% CI]
**Phenobarbital**	1.00	0.78 [0.31–1.94]	0.33 [0.07–0.45]
**Valproic acid**	1.00	0.43 [0.05–3.79]	NA
**Carbamazepine**	1.00	0.82 [0.29–2.35]	1.28 [0.35–4.66]
**Phenytoin**	1.00	NA	NA
**Diazepam**	1.00	NA	NA
**Lamotrigine**	1.00	2.80 [0.16–46.56]	21.91 [2.07–231.60]
**Carbamazepine and Phenobarbital**	1.00	0.27 [0.06–1.19]	0.56 [0.16–1.85]
**Carbamazepine and Diazepam**	1.00	NA	NA
**Phenobarbital and Valproic acid**	1.00	2.21 [0.48–10.09]	0.72 [0.13–3.95]
**Phenobarbital and Diazepam**	1.00	5.75 [0.61–53.43]	12.04 [1.43–101.47]
**Lamotrigine and Valproic acid**	1.00	3.13 [0.30–32.48]	1.53 [0.13–17.97]

source: author’s own production

Some of seizures types was used as reference.

NA: analysis could not be performed.

In the logistic regression analysis of PWWE who were prescribed drugs during monotherapy and polytherapy and who did or did not use ASM, no statistically significant association was observed in adverse obstetric and neonatal outcomes (Table 6) (S2–S4 Tables).

**Table 6:** Univariable and multivariable adjusted logistic regression analysis of obstetric and neonatal outcomes between PWWE using and not using ASM, and ASM in monotherapy and polytherapy (n = 229).

Dependent Variables	Univariable analysis		Analysis with adjustment for age, type of delivery, marital status, place of birth		Univariable analysis		Analysis with adjustment for age, type of delivery, marital status, place of birth	
	No ASM use	ASM use	No ASM use	ASM use	Monotherapy	Polytherapy	Monotherapy	Polytherapy
	OR [95% CI]	OR [95% CI]	OR [95% CI]	OR [95% CI]	OR [95% CI]	OR [95% CI]	OR [95% CI]	OR [95% CI]
**PrH**	1.00	0.96 [0.37–2.53]	1.00	0.83 [0.30–2.28]	1.00	0.87 [0.41–1.82]	1.00	0.98 [0.46–2.08]
**Vaginal bleeding**	1.00	NA	1.00	NA	1.00	0.21 [0.04–0.98]	1.00	0.98 [0.46–2.08]
**Preeclampsia**	1.00	NA	1.00	NA	1.00	0.29 [0.06–1.40]	1.00	1.54 [0.20–11.47]
**Eclampsia**	1.00	NA	1.00	NA	1.00	1.05 [0.22–4.84]	1.00	1.39 [0.65–2.99]
**Miscarriage**	1.00	1.47 [0.53–4.06]	1.00	1.65 [0.51–5.33]	1.00	1.18 [0.56–2.21]	1.00	0.29 [0.06–1.42]
**MCM**	1.00	NA	1.00	NA	1.00	1.18 [0.56–2.21]	1.00	1.05 [0.22–5.04]
**Premature delivery <37 weeks**	1.00	1.06 [0.42–2.62]	1.00	1.02 [0.39–2.67]	1.00	1.19 [0.57–2.49]	1.00	0.09 [0.01–0.79]
**Low birth weight**	1.00	0.98 [0.37–2.57]	1.00	0.92 [0.33–2.57]	1.00	1.41 [0.19–10.23]	1.00	0.84 [0.38–1.84]

source: author’s own production

Reference: pregnant women with epilepsy not taking AEDs.

NA: analysis could not be performed. *P < 0.05.

## Discussion

Recent studies have revealed that PWWE have a higher risk of obstetric complications, especially mild preeclampsia, than PWNE [5, 6]. The findings of our study compared PWWE/PWNE and proved that women with epilepsy had higher chances of developing complications, such as PrH, preeclampsia, vaginal bleeding, oligohydramnios, polyhydramnios, miscarriage, and stillbirth. These results are consistent with an American retrospective cohort study from 2007 to 2011 conducted in 20% of community hospitals that observed a statistically significant association between PrH (adjusted OR = 1.30, 95% CI = 1.27–1.33), preeclampsia (adjusted OR = 1.59, 95% CI = 1.54–1.63), and stillbirth (adjusted OR = 1.27, 95% CI = 1.17–1.38) [9]. A population-based cohort study in Sweden from 1997 to 2011 observed a statistically significant association between preeclampsia (adjusted OR = 1.24, 95% CI = 1.07–1.43) and stillbirth (adjusted OR = 1.55, 95% CI = 1.05–2.30) [23]. Following the same rationale, two Norwegian population-based cohort studies also compared PWWE/PWNE; their findings were similar to those of our study in terms of mild preeclampsia (3.6% and 3.8%, respectively) [5, 6].

Regarding the delivery, our study also found a high rate of cesarean delivery, which was consistent with studies conducted in the US and China that found PWWE were more likely to have cesarean deliveries than PWNE (40.5% vs. 33.1% and 85.3% vs. 50.3%, respectively) [9, 24]. In contrast to our results (34%), European studies have reported lower rates of cesarean delivery in PWWE (18.8%,34%) [6, 15]. Additionally, two studies, one conducted in Sri Lanka and another in Merseyside/Manchester, reported a higher incidence of vaginal deliveries at 73.3% and 63%, respectively [25, 26]. More than half of the patients (51.2%) in a longitudinal cohort study composed of 90 consecutive pregnant women, who were under regular clinical follow-up between January 2005 and January 2018 in an outpatient clinic of refractory epilepsy of a university-affiliated tertiary referral hospital in São Paulo, Brazil, underwent cesarean delivery [27]. In a retrospective study in Maringá, Brazil, there was also a very high cesarean delivery rate at 72.9% [28]. Comparing women in Brazil, in general, cesarean delivery rates are variable but markedly elevated; in some regions, it can reach a prevalence of 53% [29]. In Alagoas, the cesarean delivery rates reached 53%, according to census 2021 in Brazil [21]. A retrospective cohort study on PWWE from Alagoas found a positively association with cesarean delivery [30]. Similar results were observed in a retrospective cohort study in the Turkish population with 154 PWWE and 462 controls, a cross-sectional study in Poland, and a retrospective cohort in 20% of all US hospitals; cesarean delivery was higher in PWWE at 85.7%, 49.7%, and 40.5%, respectively [9, 31, 32]. However, our study’s prospective cohort rate was higher in PWWE than in PWNE for cesarean delivery and preterm birth; however, it was not significant.

Our study showed that most PWWE used ASM for pharmacological treatment of epilepsy during pregnancy. The Australian Antiepileptic Drugs in Pregnancy Registry, conducted over the 20-year period from 1998 to 2018, showed similar results; out of the 2,148 pregnancies evaluated, 1,972 (91.8%) involved ASM use, while 176 (8.2%) women were not exposed to pharmacological treatment in the first trimester of pregnancy [33]. In contrast, the UK Epilepsy and Pregnancy Register (UKIEPR) whose prospective cohort study was conducted between December 1996 and August 2016, analyzed the outcomes of 9,247 pregnancies, of which 6,785 (73.4%) involved ASM use in monotherapy, 1,858 (20.1%) involved polytherapy regimens, and 604 (6.5%) did not involve ASM use [34].

The use of older ASM is not advised during pregnancy; however, these ASMs may be indispensable for seizure control in women with severe epilepsy [15]. ASMs with lower rates of MCMs, such as LTG, LEV, and oxcarbazepine (OXC), are often preferred for PWWE [4]. However, in our study, the small number of PWWE using LTG and no pregnant women using OXC and LEV, revealed that the high-risk pregnancy reference centers in Alagoas did not usually prescribe these drugs (S1 Table). It is important to emphasize that these drugs are not freely distributed by the SUS in Alagoas, a state characterized by a socio-demographic profile of vulnerability, which may have influenced the low rate of prescription of these drugs by the neurologists in these centers.

CBZ, the second most prescribed drug in this study, might be associated with a higher risk of teratogenicity. CBZ has teratogenic capacity but to a lesser extent than valproic acid (VPA) and TPM [10]. In our study, a small number of PWWE used CBZ; however, no association between this drug and neonatal outcomes was found (OR = 1.1, 95% CI = 0.7–1.7) (S3 Table). These results corroborated the Swedish Cohort study from 1996 to 2013, which found no statistically significant association between CBZ and LTG use, preterm delivery < 37 weeks, and low birth weight (OR = 1.3, 95% CI = 0.7–2.6) [11].

The 20-year Australian registry regarding ASM as well as a systematic review and meta-analysis by Viale et al. (2015), found an association between spontaneous abortions in women with epilepsy who were prescribed ASM, which differs from our study that found no statistically significant evidence for this association (OR = 1.45, 95% CI = 1.12–1.87 and OR = 1.54, 95% CI = 1.02–2.32, respectively) [33, 35].

De Lima Leite et al. (2022), who used the same population cohort as that of our study, found a statistically significant difference in the rates of miscarriage between PWWE/PWNE; however, their study found no association between this outcome and the use or non-use of ASM [30]. Therefore, the cause of miscarriage in PWWE in this study was epilepsy.

In terms of seizure types, GTCSs are associated with risk to the fetus and pregnant women. GTCSs are more worrisome and have been reported to cause several complications, including prenatal hypoxia and ischemia in areas of the brain, placental infarction, and intrauterine intracranial hemorrhage with fetal death. Other epileptic seizures are probably less harmful but may be associated with intrauterine growth retardation and premature delivery [25]. In our study, we observed an association between GTCS and stillbirth. In Battino’s study, among 33.4% of PWWE, 15.2% had GTCSs and 21% had status epilepticus; one perinatal death was documented and no maternal deaths were noted [36]. Our study also did not document any maternal deaths, and with respect to status epilepticus, we found almost twice as many cases as those in Battino’s study, with 24% of PWWE experiencing GTCS and 52% experiencing status epilepticus. This result was higher than that found in EURAP, with only 36 GTCS and 21 status epilepticus cases, respectively [6–36]. This suggests that previous studies have reported confusing data, which were all obtained from high-income countries; therefore, they differ from data obtained from countries with more limited healthcare resources, such as those reported in our study, including the use of older ASMs that probably do not control seizures.

Finally, it should be noted that half the population did not plan pregnancies, and MCM caused by folate deficiency can occur within the first 25 days of conception (period during which pregnancy is often unknown); therefore, supplementation with folic acid (AF) should be automatically prescribed in women of childbearing age who use MAC [37]. Although some ASMs interfere with folic acid, data related to the effects of folic acid supplementation on pregnancy outcomes in women with epilepsy remain inconclusive [38]. Reports from prospective epilepsy pregnancy registries in the UK, Poland, and EURAP showed no association between folic acid use and a lower risk of MCM [39–41]. A Finnish population-based cohort study showed better results with folic acid supplementation in 43.6% of PWWE [7]. In our study, folic acid and ferrous sulfate were prescribed for a small number of PWWE, which may be related to the risk of miscarriage. These findings of miscarriage are similar to those by Asadi et al. (OR = 2.6, 95% CI = 1.2–5.6) [38]. The recent study of the effects of antiepileptic drugs on neurodevelopment (NEAD), a prospective observational investigation enrolled from October 1999 to February 2004 in 25 epilepsy centers in the US and UK, showed positive associations between periconceptional folate exposure and improved neurodevelopmental scores in a series of cognitive variables in children’s MCE who took MACs [42].

## Conclusions

Adverse obstetric and neonatal outcomes were strongly associated with PWWE, particularly in those with GTCS and status epilepticus. PB was the most used drug in this study. The probable cause of these adverse outcomes was the use of inappropriate ASM for PWWE. It is noteworthy that, in view of our results, it was not possible to identify whether the adverse obstetric and neonatal outcomes were due to epilepsy or use of ASM. Data from previous studies is confusing and comes from high-income countries, whereas there is a lack of relevant data from countries with limited healthcare resources. Therefore, studies with larger sample sizes and prospective follow-up of PWWE are necessary.

## Limitations and future perspectives

The limitation of this study was the incomplete data collected from the physical and electronic medical records. In future, we will study a prospective cohort that will help validate the results obtained from this retrospective cohort. Knowledge and understanding of the complete socio-demographic context as well as obstetric and neonatal outcomes and seizures controls in these women would help promote public policies to improve the quality of life of this population. The results revealed that the high-risk pregnancy reference centers in Alagoas did not usually prescribe new generation ASMs. It is important to emphasize that the cost of newer ASMs increases overall treatment cost; they are not yet freely distributed by the government in Alagoas, a state characterized by a vulnerable socio-demographic profile. These may have influenced the low rate of prescription of these drugs by the neurologists in these centers. These results demonstrated the need to create public policies to facilitate the distribution of appropriate ASMs for this population.

## Supporting information

S1 Table Profile of ASM used in pregnant women with epilepsy (PWWE). (PDF)

S2 Table Regression analysis between neonate outcomes and ASM used in monotherapy. (PDF)

S3 Table Regression analysis between obstetric and neonatal outcomes and ASM in polytherapy. (PDF)

S4 Table Univariable and multivariable adjusted logistic regression analysis of obstetric and neonatal outcomes between PWWE using and not using ASM, and ASM in monotherapy and polytherapy (n = 229). (PDF)

S1 Data (XLSX)
