# Supplementary material for: Obstetric and neonatal outcomes, antiseizure medication profile, and seizure types in pregnant women in a vulnerability state from Brazil
Source: PLoS One. 2024 Apr 1;19(4):e0291190. doi: 10.1371/journal.pone.0291190 (PMC10984515; doi:10.1371/journal.pone.0291190)
Supplement: S1 Table — (PDF) [file pone.0291190.s001.pdf]

S1Table. Profile of ASM used in Pregnant Women With Epilepsy (PWWE) (n=229)

Drug Treatment	N	%
**Oxcarbazepine Levetiracetan**		
**No**	229	100,0
**Others drugs**		
**yes**	26	11,1
**no**	208	88,9
**Missing data**	4	1,7
**Carbamazepine, Phenobarbital and Phenytoin**	2	1,0
no	195	99,0
total	197	100,0
**Carbamazepine and Phenobarbital**		
yes	18	9,1
no	178	90,9
total	197	100,0
**Carbamazepine and Phenytoin**		
yes	4	2,0
no	193	98,0
total	197	100,0
**Carbamazepine and Diazepam**		
yes	13	6,6
No	184	93,4
total	197	100,0
**Phenytoin and Diazepam**		

Drug Treatment	N	%
yes	6	3,0
no	191	97,0
total	197	100,0
**Phenobarbital and Diazepam**		
yes	17	91,4
no	180	8,6
total	197	100,0
**Valproicacid and Diazepam**		
yes	6	3,0
no	191	97,0
total	197	100,0
**Phenobarbital and Phenytoin**		
yes	13	6,6
no	184	93,4
total	197	100,0
**Phenobarbital and Valproicacid**		
yes	12	6,1
no	185	93,9
total	197	100,0
**Lamotrigine and Valproic acid**		
yes	6	3,0
no	191	97,0
total	197	100

source: author's own production
