# Supplementary material for: Obstetric and neonatal outcomes, antiseizure medication profile, and seizure types in pregnant women in a vulnerability state from Brazil
Source: PLoS One. 2024 Apr 1;19(4):e0291190. doi: 10.1371/journal.pone.0291190 (PMC10984515; doi:10.1371/journal.pone.0291190)
Supplement: S2 Table — (PDF) [file pone.0291190.s002.pdf]

S2 Table. Regression analysis between neonate outcomes and ASM used in monotherapy (n=115)

Monotherapy	Stillbirth<br>OR [IC95%]	Neonatal ICU<br>OR [IC95%]	Neonatal heart disease<br>OR [IC95%]	Premature Delivery <37weeks<br>OR [IC95%]	Lowbirth weight<br>OR [IC95%]
**Phenobarbital**	0.62 [0.12; 3.25]	NA	NA	0.60 [0.24; 1.48]	0.90 [0.33; 2.43]
**Valproicacid**	3.43 [0.34;34.22]	NA	NA	3.68 [0.69; 19.52]	NA
**Carbamazepine**	1.51 [0.26; 8.75]	NA	NA	1.24 [0.45; 3.40]	2.42 [0.86;6.77]
**Phenytoin**	NA	NA	NA	NA	NA
**Diazepam**	NA	NA	NA	NA	NA
**Lamotrigine**	NA	NA	NA	2.34 [0.37; 14.90]	NA

Source: author's own production
Reference: no outcomes presents

NA: It was not possible to perform this analysis.
