# Supplementary material for: Obstetric and neonatal outcomes, antiseizure medication profile, and seizure types in pregnant women in a vulnerability state from Brazil
Source: PLoS One. 2024 Apr 1;19(4):e0291190. doi: 10.1371/journal.pone.0291190 (PMC10984515; doi:10.1371/journal.pone.0291190)
Supplement: S3 Table — (PDF) [file pone.0291190.s003.pdf]

S3 Table. Regression analysis between obstetric and neonatal outcomes and ASM in polytherapy (N=229)

Polytherapy	PrH	Oligohydramnios	Miscarriage	Maternal ICU	Preeclampsia/ Eclampsia	Stillbirth	Neonatal heart disease	Neonatal ICU	Premature Delivery <37weeks	Low birthweight
**Carbamazepine and Phenobarbital**	0.54 [0.11; 2.67]	NA	0.60 [0.15; 2.34]	1.85 [0.54; 6.26]	NA	0.56 [0.06;5.05]	NA	1.82 [0.15; 21.33]	0.82 [0.23; 2.91]	1.17 [0.32;4.27]
**Carbamazepine and Diazepam**	0.86 [0.16; 4.40]	1.83 [0.17; 19.13]	0.23 [0.02;1.94]	2.11 [0.55; 8.00]	NA	NA	2.79 [0.23; 33.26]	NA	1.30 [0.35; 4.82]	1.85 [0.48;7.04]
**Phenobarbital and Valproic acid**	1.78 [0.41; 7.67]	2.03 [0.19; 21.31]	0.87 [0.27; 4.65]	0.80 [0.15; 4.07]	2.03 [0.19; 21.31]	NA	3.09 [0.25; 37.04]	NA	2.33 [0.64; 8.43]	3.37 [0.90;12.62]
**Phenobarbital and Diazepam**	1.05 [0.25;4.28]	NA	0.65 [0.16; 2.57]	2.04 [0.59;6.98]	1.29 [0.12; 13.26]	0.61 [0.06;5.48]	NA	1.96 [0.16; 23.09]	2.81 [0.87; 8.99]	1.30 [0.35;4.77]
**Lamotrigine and Valproic acid**	2.66 [0.43;16.22]	NA	0.64 [0.07; 5.88]	NA	4.86 [0.42; 55.71]	2.33 [0.23;23.34]	NA	7.40 [0.56; 96.26]	0.68 [0.07; 6.51]	0.91 [0.09;8.82]

Source: author's own production

NA: It was not possible to perform this analysis.
