# Supplementary material for: Obstetric and neonatal outcomes, antiseizure medication profile, and seizure types in pregnant women in a vulnerability state from Brazil
Source: PLoS One. 2024 Apr 1;19(4):e0291190. doi: 10.1371/journal.pone.0291190 (PMC10984515; doi:10.1371/journal.pone.0291190)
Supplement: S4 Table — (PDF) [file pone.0291190.s004.pdf]

S4 Table. Univariable and multivariable adjusted logistic regression analysis of obstetric and neonatal outcomes between PWWE using and not using ASM, and ASM in monotherapy and polytherapy(n=229)

**Dependent Variables**	**Univariable analysis**				**Analysis with adjustment for age, type of delivery, marital status, place of birth**			
	**Monotherapy**		**Polytherapy**		**Monotherapy**		**Polytherapy**	
	**OR**	**IC 95%**	**OR**	**IC 95%**	**OR**	**IC 95%**	**OR**	**IC 95%**
**Obstetric outcomes (PrH, vaginal bleeding, preeclampsia and eclampsia)**	1.89	0.71; 5.01	1.13	0.40; 3.19	1.90	0.68; 5.28	0.94	0.31; 2.84
**Neonatal outcomes (Stillbirth and MCM)**	1.12	0.22; 5.56	1.84	0.37; 9.06	NA	NA	NA	NA

Source: author's own production
Reference: pregnant women with epilepsy not taking ASM

NA: It was not possible to perform this analysis.
